# Fabrication, assessment, and optimization of alendronate sodium nanoemulsion-based injectable *in-situ* gel formulation for management of osteoporosis

**DOI:** 10.1080/10717544.2022.2164094

**Published:** 2023-01-01

**Authors:** Wesam H. Abdulaal, Khaled M. Hosny, Nabil A. Alhakamy, Rana B. Bakhaidar, Yasir Almuhanna, Fahad Y. Sabei, Mohammed Alissa, Mohammed Majrashi, Jawaher Abdullah Alamoudi, Mohannad S. Hazzazi, Ayman Jafer, Rasha A. Khallaf

**Affiliations:** a Department of Biochemistry, Faculty of Science, Cancer and Mutagenesis Unit, King Fahd Center for Medical Research, King Abdulaziz University, Jeddah, Saudi Arabia; b Center of Artificial Intelligence in Precision Medicines (CAIPM), King Abdulaziz University, Jeddah, Saudi Arabia; c Department of Pharmaceutics, Faculty of Pharmacy, King Abdulaziz University, Jeddah, Saudi Arabia; d Center of Excellence for Drug Research and Pharmaceutical Industries, King Abdulaziz University, Jeddah, Saudi Arabia; e Department of Medical Laboratories, College of Applied Medical Sciences, Shaqra University, Shaqra, Saudi Arabia; f Department of Pharmaceutics, College of Pharmacy, Jazan University, Jazan, Saudi Arabia; g Department of Medical Laboratory Sciences, College of Applied Medical Sciences, Prince Sattam bin Abdulaziz University, Al-Kharj, Saudi Arabia; h Department of Pharmacology, College of Medicine, University of Jeddah, Jeddah, Saudi Arabia; i Department of Pharmaceutical Sciences, College of Pharmacy, Princess Nourah bint Abdulrahman University, Riyadh, Saudi Arabia; j Department of Medical Laboratory Sciences, Faculty of Applied Medical Sciences, King Abdulaziz University, Jeddah, Saudi Arabia; k Hematology Research Unit, King Fahd Medical Research Center, King Abdulaziz University, Jeddah, Saudi Arabia; l Department of Pharmaceutics and Industrial Pharmacy, Faculty of Pharmacy, Beni-Suef University, Beni-Suef, Egypt

**Keywords:** In situ gel, alendronate sodium, nanoemulsion, PPSG, osteoporosis, design of experiment

## Abstract

Low bone mass, degeneration of bone tissue, and disruption of bone microarchitecture are all symptoms of the disease osteoporosis, which can decrease bone strength and increase the risk of fractures. The main objective of the current study was to use a phospholipid-based phase separation *in-situ* gel (PPSG) in combination with an alendronate sodium nanoemulsion (ALS-NE) to help prevent bone resorption in rats. The effect of factors such as concentrations of the ALS aqueous solution, surfactant Plurol Oleique CC 497, and Maisine CC oil on nanoemulsion characteristics such as stability index and globular size was investigated using an l-optimal coordinate exchange statistical design. Injectable PPSG with the best nanoemulsion formulation was tested for viscosity, gel strength, water absorption, and *in-vitro* ALS release. ALS retention in the rats’ muscles was measured after 30 days. The droplet size and stability index of the optimal nanoemulsion were 90 ± 2.0 nm and 85 ± 1.9%, respectively. When mixed with water, the optimal ALS-NE–loaded PPSG became viscous and achieved 36 seconds of gel strength, which was adequate for an injectable *in-situ* formulation. In comparison with the ALS solution–loaded *in-situ* gel, the newly created optimal ALS-NE–loaded PPSG produced the sustained and regulated release of ALS; hence, a higher percentage of ALS remained in rats’ muscles after 30 days. PPSG that has been loaded with an ALS-NE may therefore be a more auspicious, productive, and effective platform for osteoporosis treatment than conventional oral forms.

## 1. Introduction

Because of the reduced bone mineral density (BMD), poor bone microarchitecture/mineralization, and/or low bone strength in osteoporosis, such condition increases a patient’s risk of bone fracture (Wright et al., 2014). Until the patient presents with a low-trauma fracture of the hip, spine, proximal humerus, pelvis, or wrist, which often necessitates hospitalization, this asymptomatic ailment frequently goes untreated (Burge et al., 2007). It is more commonly associated with women, but men can also be diagnosed with osteoporosis; in fact, men make up around one in five of all Americans with low BMD or osteoporosis (Raisz, 2005). Osteoporosis is not only the leading cause of fractures in the elderly population.

Due to its impact on osteoclasts, the oral bisphosphonate alendronate sodium (ALS) prevents bone resorption. Its suppressive effect is strong and targeted (Hosny et al., 2013). All recommendations call for the use of ALS to treat osteoporosis, whether in postmenopausal women or in patients with osteoporosis brought on by glucocorticoid use (Bone et al., 2004). The mechanisms of action of ALS include an increase in bone density brought on by the inhibition of bone turnover and a 50% reduction in the risk of fractures of the spine and hip (Schnitzer et al., 2000). The low systemic bioavailability of this medication from commercially available tablets, for which the absorption rate is roughly 0.6% following oral administration, is just one of numerous difficulties with its use. The absorption is poor because the molecule’s extremely low lipophilicity and the negative charges they carry make it difficult for them to pass through the gastrointestinal tract (Hosny, 2016). Esophageal side effects such esophagitis, ulcers, bleeding, and erosions are other drawbacks to its use (Peters et al., 2001). The medicine is only given and used under stringent safeguards and with precise instructions as a result of the aforementioned difficulties. The patient should stand up for half an hour after taking the medication, swallow the tablet with a full glass of water, and refrain from taking any other medications for at least an hour following administration, according to the instructions (Yang & Owusu-Ababio, 2000). Many incidences of esophagitis have been linked to patients who did not adhere to the recommended treatment plan (Bauer et al., 2000). As a result, during the first year of therapy, up to 60% of patients stop taking the medication containing ALS (Tadrous et al., 2014).

A research group employed the enteric coated solid lipid nanoparticles (SLNs) to enhance the bioavailability of ALS and lessens the mucosal irritating impact by preventing the medication in SLNs from dissolving in the stomach and increasing the intact amount of SLNs reaching duodenum (Hosny, 2016). In order to increase the drug’s bioavailability and acceptability by biological systems, as well as to maximize the therapeutic effects of alendronate while limiting its hazardous side effects, another research group has produced alendronate-loaded CS NPs (Pathak et al., 2022). However, a new oily based formulation might be needed to further sustain the ALS release and prevent the expected burst release that usually accompanies the drug release from aqueous based formulation. Therefore, an oily based in-situ gelling system might be a useful alternative.

A type of medication delivery system that has various benefits is an *in-situ* gelling system. Under typical *in-vitro* conditions, this delivery system is in the form of a solution that is transformed into a gel. This transformation aids in achieving the desired result (Packhaeuser et al., 2004). The *in-situ* gel has been used in a number of applications, such as vaginal, nasal, ocular, intraperitoneal, injectable, and controlled release drug delivery systems (Kranz et al., 2001). Different types of polymers utilized to create the *in-situ* gel base have been reported to undergo a number of solution–gel transformations. Temperature changes from room temperature to body temperature, pH changes from the preparation pH to the biological pH, and other factors might cause the transformation (Chenite et al., 2000).

Unlike for normal gels, intramuscular injections of *in-situ* gels are simple to give. After injection, the gel expands and solidifies inside the muscle (Dimatteo et al., 2018). The gel matrix can then release a medicine in a regulated manner over a long period of time. Therefore, it has been recommended that the hydrophobic drug ALS be incorporated into the phospholipid-based phase separation gel (PPSG), which is readily injectable due to its reduced incipient viscosity and capacity to convert into an *in-situ* insert at an injection site when placed in an aqueous medium (Zhang et al., 2019). Due to their injectability and ability to generate drug depots, *in-situ* implants are promising models (Dai et al., 2009; Hatefi & Amsden, 2002; Kempe & M€ader, 2012). Additionally, due to the biocompatibility and reduced cytotoxicity of PPSGs and the ease of obtaining phospholipids, PPSGs are desirable *in-situ* implants (Helgeson et al., 2012; Vintiloiu & Leroux, 2008).

*Nanotechnology* is defined as the manipulation of materials at the nanoscale (Salem et al., 2022). Numerous nations and significant organizations have become interested in investing in the rapidly emerging industry of nanotechnology research (Ali et al., 2021; Hussein et al., 2022). Nanoscale materials have previously unheard-of physical, chemical, and biological properties that could enhance or change the characteristics of drugs (Alhakamy et al., 2022; Salem et al., 2022). For the improvement of features of medications such as solubility and stability, successive delivery paradigms called nanoemulsions (NEs) are useful (Rizg et al., 2021). They are primarily being researched as delivery platforms for various active agents since they typically consist of nanosized oil droplets, which are stabilized through an admixture of surfactants and co-surfactants (Hosny et al., 2021a). When compared with other dosage forms, NEs have a number of advantages, such as 1) a high absorption rate with minimal divergences; 2) activity that reduces antioxidants and hydrolysis; 3) an affinity for lipophilic and hydrophilic drugs; 4) increased bioavailability; 5) reduced adverse effects, cytotoxicity, and inflammation, particularly when administered to the skin or mucus membranes; and 6) prolonged drug release. The nano-size of the globules is credited with some of these benefits, while the surfactant and co-surfactant components of NEs may be responsible for others (Hosny et al., 2021b, 2021c).

The main objectives of a statistical experimental design are to define interactions between variables, obtain the greatest amount of information from the fewest trials possible, and identify the causes of experimental errors (Alkhalidi et al., 2018). Such designs also require accurate planning and adherence to statistical formulas, which is a benefit. The accuracy of the study aims and investigations that must be carried out to achieve those goals must be pursued rigorously by investigators. The optimal composition of a formulation and the methods for developing it on a wide scale can also be predicted by the design (Hosny et al., 2020). In a way, experimental designs are economical because they frequently present the best option for the targeted formulation (Salem et al., 2020). The chief objective of this research was to fabricate an innovative injectable oily *in-situ* formulation loaded with ALS to provide a prolonged-release intramuscular depot of the drug, which would be given four times a year. Such a paradigm is expected to raise ALS bioavailability and enhance patient adherence.

## 2. Materials and methods

### 2.1. Materials

ALS was acquired from Creative Biomart (Shirley, NY, USA). Phospholipid S100 was secured from Lipoid GmbH (Ludwigshafen am Rhein, Germany). Plurol Oleique CC 497 ® (polyglyceryl-3 dioleate), Maisine CC ® (glyceryl monolinoleate), and Peceol ® (glycerol monooleate) were kind gifts from Gattefosse (Saint-Priest, France). Absolute ethanol was procured from the Sigma-Aldrich Corporation (St. Louis, MO, USA). All other reagents and chemicals were of HPLC grade and employed with no additional purification.

### 2.2. Preparation and optimization of ALS-loaded NE as per mixture design

The I-optimal coordinate-exchange quadratic mixture design utilizing Design-Expert software was used to create ALS-loaded NEs (version 13.0.7.0, Stat-Ease, Inc., Minneapolis, MN, USA). The percentages of ALS aqueous solution (factor A), surfactant Plurol Oleique CC 497 ® (factor B), and Maisine CC ® (factor C) ranged from 0.1 to 0.2, 0.3 to 0.5, and 0.3 to 0.6, respectively. The experimental design evaluated the effects of these parameters. The three independent variables were combined in a variety of ratios, but the overall concentration remained constant at 100%. The average size of droplets (Y_1_) and the average stability index (Y_2_) were chosen as the dependent variables. We generated 16 formulations at random. Table 1 lists the variables and their corresponding levels for each formulation. Regression equations and statistical analysis techniques of Design-Expert software were used to further analyze the relationship between independent factors and dependent variables. All created nanodispersions were assessed for their ability to emulsify and their appearance. The formulation chosen as the best one for creating an ALS-NE *in-situ* gel was the one with the smallest droplet size and highest stability index.

**Table 1.** Statistical design for NE formulation (independent variables, measured responses and their constraints).

Component	Level		Response	Aim
	Low	High		
Alendronate sodium aqueous solution level (A)	0.1	0.2	Mean globule size (Y_1_) Stability index (Y_2_)	Minimize Maximize
Plurol Oleique CC ®surfactant level (B)	0.3	0.5		
Maisine CC ® oil level (C)	0.3	0.6		

### 2.3. Preparation of ALS-NEs

An aqueous solution of ALS with a concentration of 100 g/ml was combined with Plurol Oleique CC ® emulsifier to create an NE. To create a water-in-oil emulsion, the sparse aqueous phase was mixed with the external oily phase (i.e., Maisine CC ® oil) with dropwise additions, and a probe sonicator mixed these phases for 3 minutes with a 40% amplitude in 5-second pulses (i.e., 5 seconds off and 5 seconds on) (Hosny et al., 2021c).

### 2.4. Assessment of ALS-NEs

#### 2.4.1. Emulsification ability

A visual examination was done of the rapid emulsification capacity and transparency of the ALS-NEs to assess the NE competency (Rizg et al., 2021).

#### 2.4.2. Droplet size assessment of the ALS-NEs

To avoid the multiple scattering influence and reduce the impact of the NE’s viscosity, certain volumes of NE formulations were diluted using Peceol (ratio 1:10 v/v). The targeted samples were subjected to the dynamic light-scattering method (Zetatrac, Microtrac, Montgomeryville, PA, USA) in order to quantify the size of the NEs’ droplets and the NEs’ polydispersity indexes (PDIs). Assessments were done in triplicate (Rizg et al., 2021).

#### 2.4.3. Stability studies

All NE formulations were subjected to a heat–cool stability valuation in the first step. Formulations were stored at 4 °C for 48 hours, followed by another 48 hours at 40 °C. This cycle was repeated three times, and then the formulations were visually examined for indications of instability. The thermodynamic stability of the investigated NEs was confirmed using the freeze–thaw accelerated stability evaluation at various temperatures (Rizg et al., 2021). In this investigation, the samples underwent three successive freeze–thaw cycles once the droplet size was determined (freezing at −25 ^0 ^C for approximately 24 hours and thawing at room temperature for another 24 hours). The sizes of the droplets were calculated following each cycle. The stability index of the formulations was assessed by comparing the final droplet size with the initial one, applying the equation below (Hosny et al., 2021a):

(1) Stability index = ([Initial size−Change in size]/Initial size)×100

### 2.5. Optimization procedure of ALS-NEs

The ALS-NEs were optimized in accordance with the stated objectives in Table 1. The ALS aqueous solution (A), Plurol Oleique CC ® (B), and Maisine CC ® oil (C) were used for the optimization at levels of 0.1, 0.5, and 0.4, respectively. The best formulation was the one with the smallest droplet size and the highest stability index.

### 2.6. Development and assessment of the optimized ALS-NE–loaded in-situ gel

The best ALS-NE dispersion was chosen, and a phospholipid-based phase separation *in-situ* gel (PPSG) method was used to create the NE *in-situ* gel. Phospholipid S100 and Peceol ® were mixed in a 2:1 (w/w) ratio to create the gel base, which was added to 100% ethanol and stirred with a magnet at 800 rpm for 2 hours at 25 °C to create a clear, uniform mixture. To form an ALS-NE–loaded *in-situ* gel (10 g/ml), the optimal ALS-NE was dispersed in the established *in-situ* gel base and sonicated for 10 minutes using a water bath sonicator (Hassan et al., 2016).

#### 2.6.1. Viscosity measurement

The optimal loaded *in-situ* gel solution was thoroughly mixed with phosphate buffered saline (PBS 30%, 0.01 M, pH 7.4) to produce the appropriate transitional gel. Before and after the addition of the PBS, the viscosity was calculated at room temperature utilizing a DV-2 T rotary viscometer (Brookfield Engineering, Middleboro, MA, USA) fitted with a No. 18 spindle with the speed set at 20 rpm (Abdelbary et al., 2017).

#### 2.6.2. Measurement of gel strength

An optimal formulation–loaded *in-situ* gel sample weighing 5 g was converted to a gel by mixing it with 1 ml of PBS (0.01 M, pH 7.4), as explained above. A 3.5-g weight having a 0.7-cm diameter was then placed on top of the gel formulation. The time, in seconds, necessary for the weight to sink 3 cm into the formulation was used to evaluate the gel strength, which predicted the *in-situ* gel viscosity considering physiological conditions (Hassan et al., 2016).

#### 2.6.3. Water absorption

A dialysis bag containing approximately 1 g of *in-situ* gel loaded with the optimal formulation was dipped into 400 ml of PBS while being magnetically stirred at 200 rpm. The gel was carefully removed and weighed at specific time periods (i.e., 5, 15, 30, 60, 120, 240, and 480 minutes). The gel was then dissolved by ultrasound with the addition of anhydrous methanol, which was five times the weight of the investigated NEs. Utilizing a V20 Volumetric Karl Fischer moisture analyzer (Mettler Toledo, Columbus, OH, USA) at an injection volume of 200 µl, the water content of the methanol–gel solution was determined. Based on the dilution factor, the concentration of water could be computed. Additionally, the proportion of water content to the dialyzed gel weight could be calculated (Hoshino et al., 2019).

#### 2.6.4. *In-vitro* release study

A dialysis bag with a 5-ml sample of PPSG containing the optimized ALS-NE was placed in a vessel with 100 ml of PBS (pH 6.8 at 37 1 0 C). For 120 hours, the solution was stirred at 50 rpm. At certain intervals, samples were removed and their ALS content was calculated using a spectrophotometer set at 265 nm. In this experiment, the ALS aqueous dispersion *in-situ* gel served as a control (Abdelbary et al., 2017).

#### 2.6.5. *In-vivo* study

For each of three distinct formulations (i.e., the *in-situ* gel loaded with the optimized ALS-NE, the ALS suspended in PPSG, and the ALS hydrogel developed by dispersing ALS in 1.5% hydroxypropyl cellulose solution), 0.5 ml was injected intramuscularly into three groups of 27 male rats each at random. This study was performed according to the institutional guidelines of the Animal Ethics Committee of Cairo Agriculture for Experimental Animals, Cairo, Egypt, Approval No. (107-09-22). At 1, 3, 6, 12, and 24 hours and 3, 7, 14, and 30 days after injection, rats were slaughtered. The tissue covering the leftover gel in the muscle was removed. The clean gels were weighed and the weights recorded, and the gels were dissolved by ultrasound with anhydrous methanol five times their weight. Then, the ALS content was determined *in vitro* using the procedures outlined earlier. In the end, we could calculate the *in-vivo* release rate of the ALS from the manufactured *in-situ* gel loaded with ALS-NE by calculating the percentage of ALS released *in vivo* and generating the matching percentage content–time curve.

## 3. Results and discussion

### 3.1. Assessment of the ALS-loaded NEs

The created nanodispersions were discovered to be uniform, nearly clear in appearance, and devoid of deposits.

#### 3.1.1. Evaluation of ALS-loaded NE droplet size

As shown in Table 2, the manufactured NEs had droplet sizes of 96 to 311 nm. They had PDI values ranging from 0.15 to 0.30. These results demonstrated the ALS-loaded NEs’ adequate stability, homogeneity, and size distribution. It was decided to analyze the droplet size data using a quadratic model of polynomial analysis since it showed the best considerable average squared value overriding the residual error (*p* < .0001). The selected model could adequately account for the substantial effects of the levels of the ALS aqueous solution (A), Plurol Oleique CC ® (B), and Maisine CC ®oil (C) on the ALS-NEs’ droplet size, according to the proposed statistical design. The adjusted R^2^ value for the model was 0.9957, which was somewhat in line with the expected R^2^ value of 0.9912. The analysis of variance (ANOVA) of the data produced the following equation.

(2) Mean globule size = +259.50 A + 79.10 B + 229.35 C – 2.41 AB + 325.25 AC – 163.27 BC

**Table 2.** The proposed l-optimal exchange co-ordinate experimental design and measured responses of ALS-NEs.

Run	A: ALS solution level	B: Plurol Oleique CC® surfactant level	C: Maisine CC ® oil level	Y_1_: Mean globule size (nm)	Y_2_: Stability index (%)	PDI
1	0.147	0.494	0.358	122 ± 2.5	81 ± 0.5	0.21
2	0.146	0.419	0.434	170 ± 1.9	79 ± 1.0	0.25
3	0.200	0.444	0.355	176 ± 3.3	77 ± 0.7	0.15
4	0.150	0.400	0.450	180 ± 4.5	78 ± 1.2	0.19
5	0.146	0.419	0.434	167 ± 2.1	78 ± 0.4	0.22
6	0.149	0.300	0.550	280 ± 5.4	72 ± 1.5	0.17
7	0.192	0.500	0.307	138 ± 2.9	79 ± 0.5	0.27
8	0.100	0.400	0.499	140 ± 2.5	79 ± 0.8	0.30
9	0.100	0.326	0.573	200 ± 4.2	77 ± 1.0	0.22
10	0.192	0.350	0.456	249 ± 3.5	75 ± 0.4	0.19
11	0.144	0.350	0.504	231 ± 4.9	75 ± 0.3	0.28
12	0.100	0.500	0.400	96 ± 1.5	84 ± 1.6	0.16
13	0.200	0.300	0.500	311 ± 4.1	72 ± 0.9	0.19
14	0.200	0.395	0.404	210 ± 3.2	75 ± 0.5	0.24
15	0.100	0.326	0.573	205 ± 4.4	78 ± 0.6	0.15
16	0.149	0.300	0.550	278 ± 1.8	73 ± 0.7	0.23

**Figure 1.**
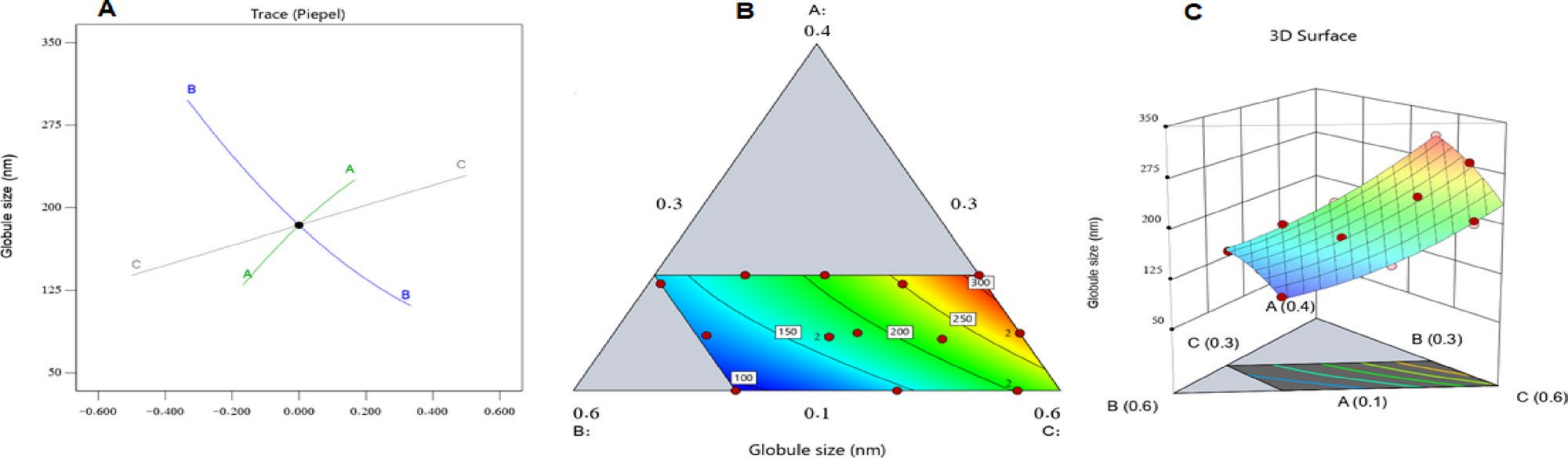
Perturbation graph (a), contour plot (b), and 3 D surface plot (c) illustrating the influence of different factors on the droplet size (Y_1_) of various ALS-NE formulations.

Figure 1 displays the perturbation, 3 D surface, and contour plots that demonstrated how the conditions under investigation affected the size of the ALS-NE droplets. As seen, factor A (i.e., the ALS aqueous solution level) had a favorable impact on the droplet size; hence, it was anticipated that the size would grow as the concentration of the ALS aqueous solution increased. Such a finding might be generated by increasing the concentration of the drug’s aqueous solution, which would increase the expected size of the internal phase droplets (Hassan et al., 2015). The amphiphilic character of factor B explained why increasing the surfactant level (i.e., factor B) resulted in a matching decrease in droplet size. Since Plurol Oleique CC ® is a nonionic surfactant, it can lower the interfacial tension between the organic and aqueous phases, which in turn would cause the droplets of the NE’s internal phase to become smaller (Hosny et al., 2020). Additionally, the stiffness of the surfactant film that forms around the NE droplets would increase due to the increase in surfactant content, and this would improve the formulation’s stability and prevent aggregation (Hosny et al., 2021a). The concentration of Maisine CC ® oil, or factor C, led to a significant increase in the droplet size in the interim. Such a discovery could be attributed to the drop in the amphiphilic compound level that would occur as the level of oil increased. This would reduce the surfactant’s ability to decrease the droplet’s size and stability, which would produce more aggregation and bigger droplets (Hosny et al., 2020).

#### 3.1.2. ALS-NE stability index assessment

According to Table 2, the stability indices of the ALS-loaded NEs ranged from 72% to 84%. The stability index data were analyzed using a linear polynomial analysis paradigm since it provided the highest considerable average squared value that exceeded the residual error (*p* = .0001). The chosen mathematical design demonstrated the model’s capacity to account for the considerable leverage of the ALS aqueous solution level (A), Plurol Oleique CC ® level (B), and Maisine CC ® level (C) on the stability index of the ALS-NEs. The adjusted R^2^ value for the suggested model was 0.9358, which was quite similar to the predicted R^2^ value of 0.9158. The stability index data underwent an ANOVA analysis, yielding the equation shown below:

(3) Stability Index = +62.68A + 87.46 B + 75.75 C

**Figure 2.**
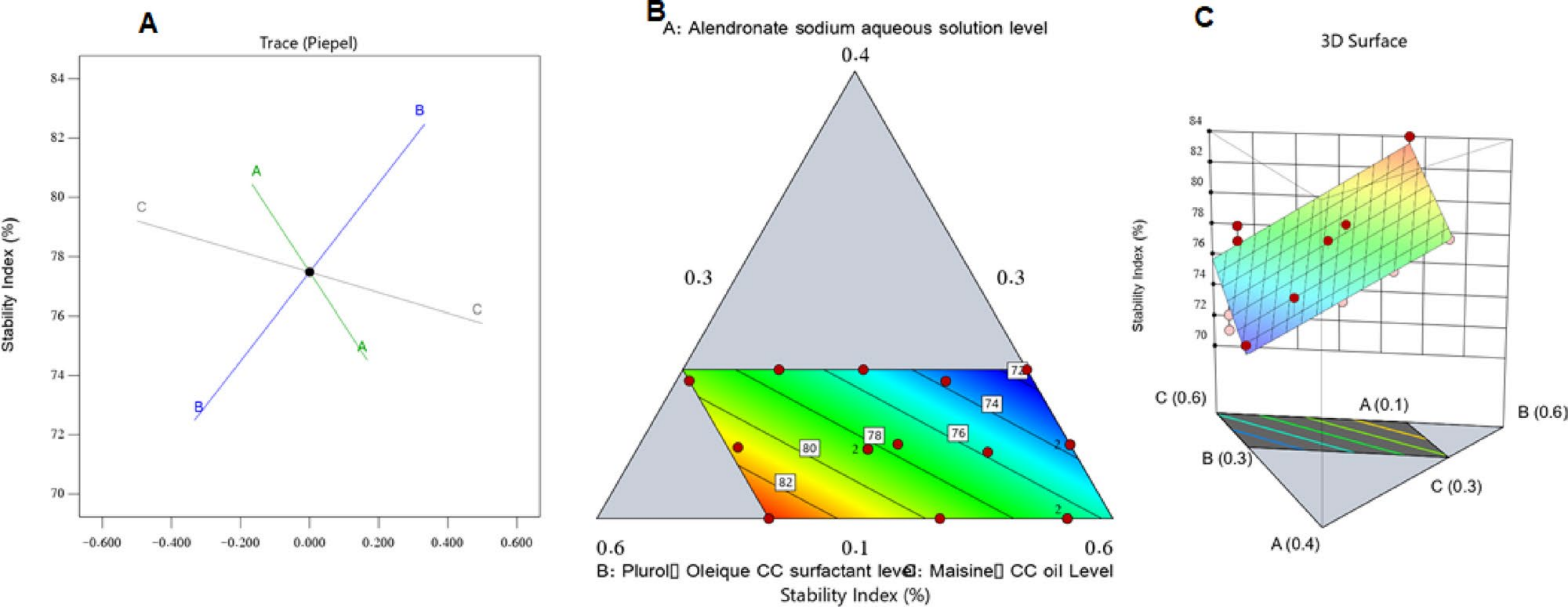
Perturbation graph (a), contour plot (b), and 3 D surface plot (c) illustrating the influence of different factors on the stability index (Y_1_) of various ALS-NE formulations.

Figure 2 shows the 3 D surface, perturbation, and contour plots of the influence of the elements under investigation on the stability index of the ALS-NEs. As shown, the level of ALS aqueous solution (factor A) had a negative effect on the stability index; as a result, increasing the concentration of the ALS aqueous solution would make the NE less stable. The projected increase in NE droplet size with an increased concentration of the drug’s aqueous solution could be cited as the cause of this phenomenon. Therefore, in accordance with Stock’s law, the interior phase globules became sedimented more quickly, decreasing the stability (Hosny et al., 2021a). In the meantime, the stability index increased in tandem with the increase in the surfactant level (factor B). The associated reduction in droplet size, which would slow the globules’ rate of sedimentation, can be used to explain these findings. Furthermore, when the quantity of surfactant increases, a firmer surfactant film forms around the internal phase droplets, improving their stability and preventing aggregation (Hosny et al., 2021b). Parallel to this, a significant drop in the stability index was provided by the increase in the Maisine CC ® oil level (factor C). This decline in NE stability may be caused by the drop in the level of the amphiphilic compound that occurred with an increase in the oil level; this would reduce the surfactant’s ability to reduce the droplet size and increase the stability, which would result in more aggregation and larger droplets with a quicker deposition rate and, consequently, a decline in stability (Hosny et al., 2021c).

### 3.2. Optimization and evaluation of NE formulations

**Figure 3.**
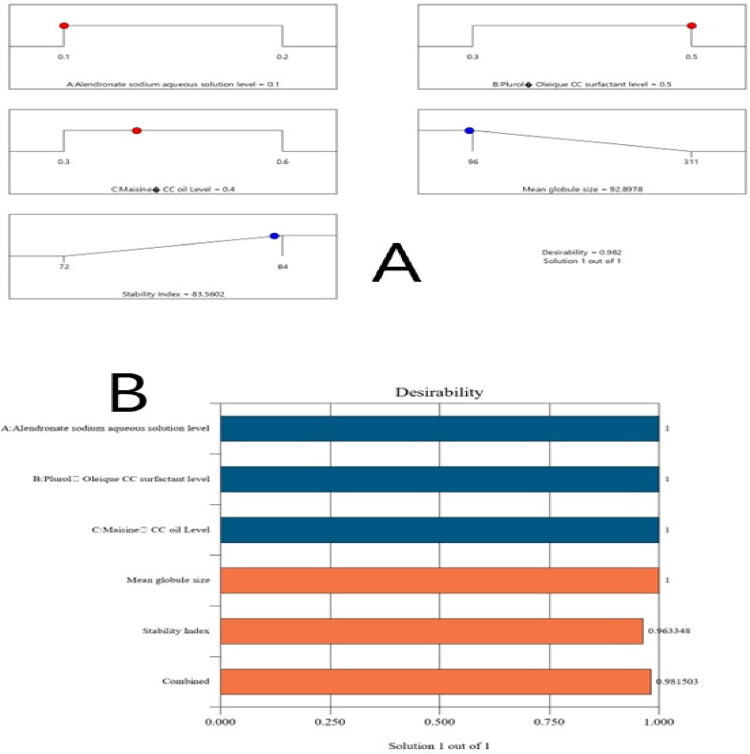
Desirability ramp and bar chart for optimization process. (a) The desirability ramp illustrated the levels of studied factors and anticipated estimations for dependent variables of the optimized formulation. (b) The bar chart presents the desirability assessments for the collective responses.

Following the aforementioned research, the optimal NE formulation was identified. The chosen design proposed many levels of combinations of the components that were evaluated. With a desirability value of 0.982, the ideal formulation was an ALS aqueous solution level of 0.1, Plurol Oleique CC ® level of 0.5, and Maisine CC ® oil level of 0.4. The created optimized ALS-NE had a droplet size of 90 ± 2.0 nm and a stability index of 85 ± 1.9%. In terms of droplet size and stability index, the results were quite near to the foreseeable merits of the same dependent variables, which were 92.8 nm and 83.5%. The bar chart and desirability ramp for the best formulation are shown in Figure 3 for various levels of independent components and anticipated measured responses.

### 3.3. Depiction of the optimal ALS-NE–loaded PPSG

#### 3.3.1. Evaluation of viscosity

Three measurements of the PPSG sample’s viscosity were taken prior to and following gelation. Before gelation, the mean viscosity was 190 ± 55 Cp, and after gelation, it was 639 ± 37 Cp. These outcomes supported the produced formulation’s *in-situ* gelation property. It is thought that solvent exchange mechanism changed the viscosity of the formulation as PBS replaced the formulation’s ethanol and caused gelation. This technique mimics what is anticipated to happen *in vivo* when a formulation is injected into the muscles, and similar findings have been previously reported (Srichan & Phaechamud, 2017).

#### 3.3.2. Measurement of gel strength

A crucial aspect of the research on creating an injectable PPSG is the gel strength. With no anticipated seepage from the injection site, a high gel strength facilitates the simple insertion of an active-component solution. Any gel formulation must have adequate gel strength to function. Formulations with a gel strength of less than 25 seconds may leak at the injection site because they may not have the necessary structural solidity. Gels having a strength of more than 50 seconds, on the other hand, could be overly strong and painful for patients. After the addition of 30% PBS, the gel strength of the generated samples was 36 seconds, and that is regarded as favorable for an injectable PPSG. Similar outcomes were reported in earlier published works (Hassan et al., 2016).

#### 3.3.3. Water absorption evaluation

Water and ethanol are miscible solvents and provide a solid foundation for the phase separation of an *in-situ* gel and the quick sedimentation of phospholipids. When ethanol comes into contact with an aqueous solution, it is believed to leak out of the *in-situ* gel while the aqueous medium permeates the gel base. A critical factor affecting ALS phase transition and release from the gel is the solvent diffusion rate (Hettiaratchi et al., 2018). *In-vitro* water absorption through the *in-situ* gel base was shown to be the most rapid within the first 2 hours, with water absorption exceeding 14% occurring within 2 minutes after dialysis. The evaluated gel base’s greatest water absorption percentage was 52 ± 3%.

#### 3.3.4. In-vitro ALS release estimation

**Figure 4.**
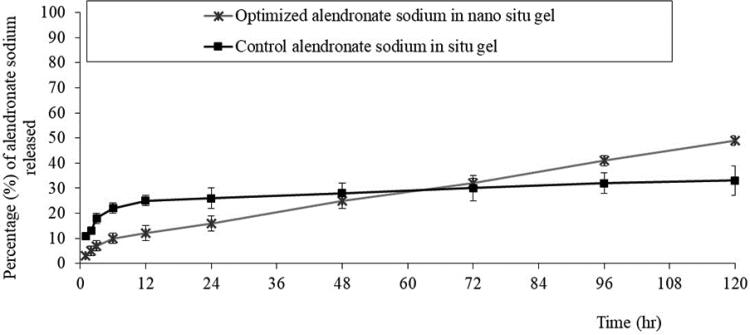
*In-vitro* release profiles of ALS aqueous solution from optimized ALS-NE–loaded PPSG and control ALS solution containing PPSG (mean ± SD, *n* = 3).

Data on the *in-vitro* release of ALS from PPSG containing the best NE formulation and a control gel made by mixing ALS solution with the gel base showed that gelation occurred within 3 hours, a release of 25 ± 4% occurred within 12 hours, and a release of 33 ± 5% occurred by the end of the experiment. In the case of the optimal gel, which gelled in approximately 1 hour, 12 ± 2% of the ALS was released within 12 hours and 50 ± 5% of the ALS was released by the end of the experiment. Figure 4 shows that the ideal formulation-loaded gel consistently released ALS over a long period of time with little release in an early burst in comparison with the control gel. The optimized formulation-loaded PPSG also produced a greater release percentage, lower standard deviation (SD), and much better release profile.

The fabricated formulation continuously delivered the medication in a sustained manner. Being in a suspended condition may be the reason behind the release behavior of ALS that displayed a release behavior close to zero-order release. Additionally, the zero-order release of PPSG can encourage sustained drug release, keep drug concentrations within the therapeutic window for a longer dosage cycle, and thus produce better anti-osteoporosis with fewer side effects. Additionally, ALS release from PPGS was extremely slow since it is a hydrophilic medication that diffuses poorly in hydrophobic materials and can only be released primarily by dissolving the carrier.

#### 3.3.5. In-vivo studies

The following was noted after analyzing the *in-vivo* experiment’s results.

Six hours was the time required for the gel to be absorbed and diffused from muscles in the group treated with the gel developed by mixing ALS in 1% hydroxypropyl cellulose solution (i.e., ≈ 40% of the ALS was released within the first hour, 95% of the ALS was released from the gel within 3 hours, and by the end of 6 hours, no gel was found at the injection site). Concerning the control group (i.e., the group that received the *in-situ* formulation containing ALS aqueous solution), only approximately 20% of the ALS releases during the first hour, 35% of the ALS was released within 3 hours, and by 6 hours, 52% of the ALS was still trapped inside the gel; this revealed that the base–gel transition took place at between 3 and 6 hours. Thereafter, the release had become regulated, and the gel was fully dissolved and absorbed by the tissues at between 7 and 14 days.

For the optimized *in-situ* gel of ALS, approximately 13% of the drug was released in the first hour and 22% of the drug in the following 3 hours, indicating that the gel transformation of the base took place at between 1 and 3 hours; after 6 hours, 70% of the drug was still entrapped in the base of the gel (i.e., 30% had been released), indicating the controlled release of the formulation. The release continued in this way for 30 days, after which 5% of the drug was still present at the site of injection.

**Figure 5.**
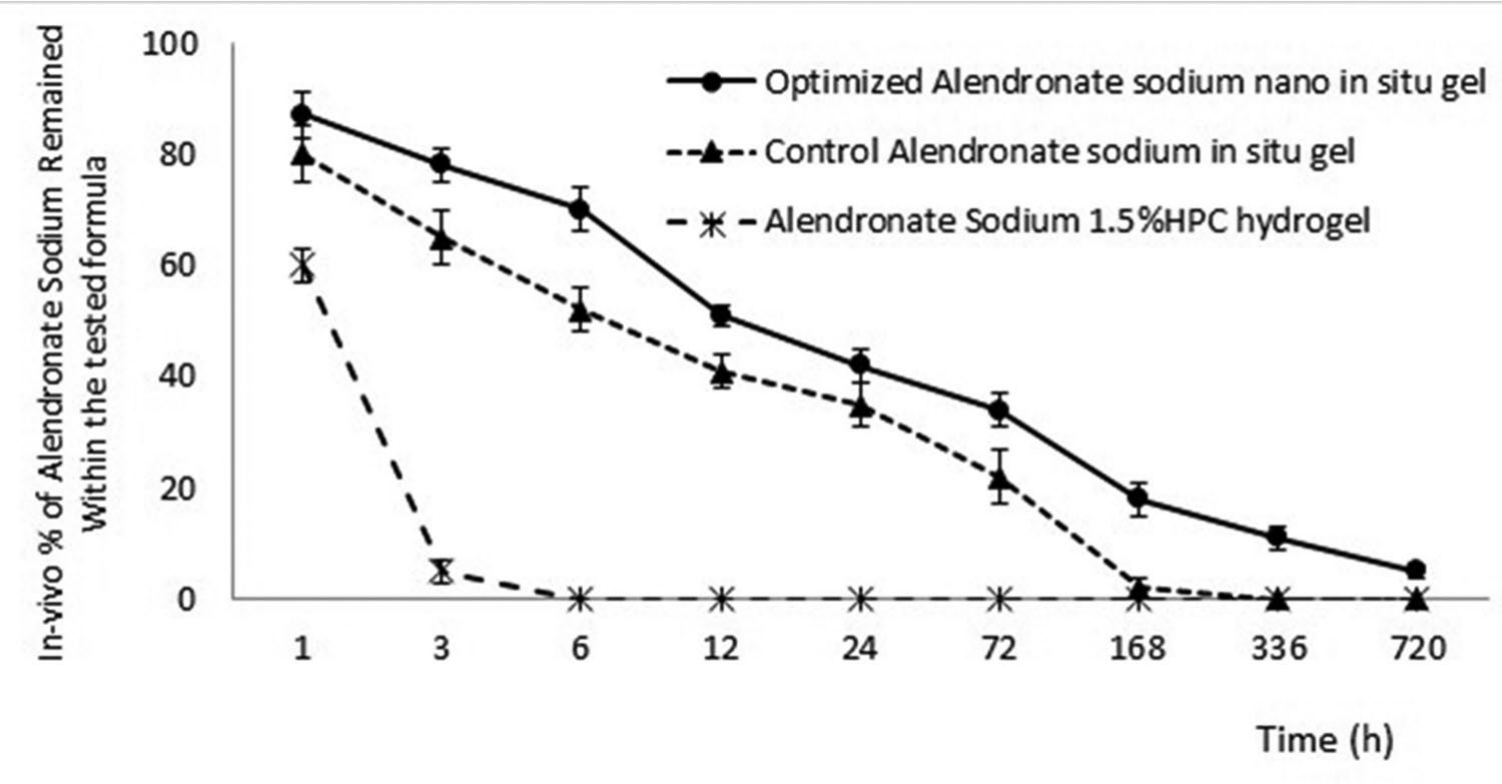
The percentage of ALS released in the muscles for various tested groups after 30 days.

The previous findings demonstrated the promising nature of the *in-situ* gel base of the optimal formulation in treating osteoporosis, one of the most common degenerative diseases, owing to its simple administration. It can be readily injected when in the liquid state and then be transformed into a gel to ensure its long-term localization at the desired site. Due to the cross-linked nature of the gel, the PPSG formulation may also offer a sustained release of ALS while in the gel state. This presents a good opportunity to maximize the influence of a dosage because it will be localized at a site for longer periods and may therefore reduce the need for larger and/or more frequent doses. Similar results have previously been documented (Camargo et al., 2013; Hassan et al., 2016). The percentage of ALS that persisted in the muscles over time (for 30 days) for various tested groups is shown in Figure 5.

## 4. Conclusion

Osteoporosis is a metabolic bone condition that increases the risk of bone fractures. A treatment for it that incorporated an NE of ALS in a PPSG *in-situ* gel seemed to be a worthwhile novel noninvasive technique for handling this disease. The animal group given the *in-situ* gel loaded with the optimal NE – which was composed of ALS aqueous solution, Plurol Oleique CC ®, and Maisine CC ® oil (C) were used for the optimization at levels of 0.1, 0.5, and 0.4, respectively- had a controlled, extended release of ALS in the muscles for 30 days. This suggested that the method was successful for administering a crucial drug simply, comfortably, and safely. This innovative technique has a wide range of potential applications in the future, particularly as a replacement for the conventional oral formulations for osteoporosis patients.
